# Intelligent Metasurface Cloak Reaches a New Plateau: AI-Assisted Surface Engineering for Self-Adaptive Supportive Invisibility

**DOI:** 10.34133/research.1130

**Published:** 2026-02-16

**Authors:** He-Xiu Xu, Zhengjie Wang, Yanzhao Wang, Yanzhang Shao, Wentao Zhang, Huanhuan Gao, Chiben Zhang, Weike Feng, Cheng-Wei Qiu, Haowei Zhang

**Affiliations:** ^1^ Electronic Science & Technology Department, Air Force Engineering University, Xi’an 710051, China.; ^2^ Department of Electrical and Computer Engineering, National University of Singapore, Singapore 117583, Singapore.

## Abstract

The past decades have witnessed the rapid development of metamaterial or metasurface cloaks, with different kinds of fascinating invisibility in phase-amplitude restoring. However, available cloaks realized thus far belong to contact-based framework; namely, they should adhere to the targets rather than being apart from each other with arbitrary distance (termed noncontact ones hereafter), which suffered from several critical shortcomings in practice. Here, we first briefly summarized some of the most important milestone works toward contact-based cloaks and then cast a holistic outlook for the conceptual noncontact stealth framework using supportive reconfigurable intelligent surface (SRIS) cloaks. Such transformative paradigm is typically composed of several SRISs deployed far away from the targets, and equipped with artificial intelligence in terms of various microcomputers, sensors, and actuators. The disruptive cloak framework full of cutting-edge techniques such as multidimensional perception, integrated radiation and scattering, and integrated sensing and communication promises to afford a brand new avenue and platform for next-generation invisibility due to their unprecedented flexibility, adaptability, and empowerment of future cloaks in interdisciplinary fields.

## Introduction

The long pursued electromagnetic (EM) invisibility has always been a fascinating dream of humanity and yielded hundreds of legends and novels until recent developments of metamaterials [1], which essentially requires complex and even singular inhomogeneous constitutive parameters of extreme anisotropy to redirect EM waves to flow around a target and thereby renders the target fully invisible [2,3]. Such stringent requirements on both the anisotropy and inhomogeneity have been leveraged to some extent by scattering cancellation mechanism with plasmonic metamaterials [4]. These cloaks intrinsically need wavelength-scale volume/thickness, such that the incoming waves could have enough long paths to interact with the structured meta-atoms and thus restore the wavefront in cloaked region. Therefore, they are usually bulky and not easy to scale up especially at high frequencies, which is very challenging in practice.

**Fig. 1.**
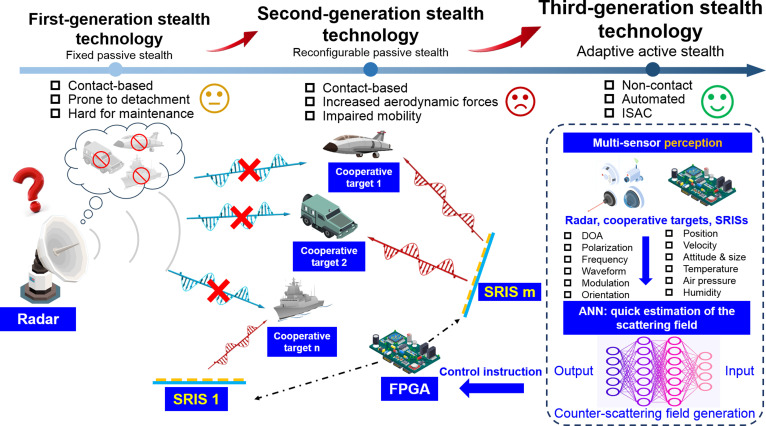
The evolutionary trajectory of 3-stage stealth technologies (top panel) along development axis, and schematic illustration of the framework and underlying operational principle of the third-generation noncontact adaptive stealth using SRIS (bottom panel). The contact-based cloaks are designated as 2 stages subjected to whether the scattering features are passively fixed or dynamically reconfigurable. For noncontact supportive stealth concept, 3 highly maneuverable cooperative targets are simultaneously concealed by several deployed RISs based on coordination mechanism when detected by a hostile radar. Most importantly, IRAS control and ISAC are 2 distinguished factors for its fully adaptive and active feature (see the “IRAS control and power management of SRIS platform” section).

As planar equivalent counterpart of metamaterials, metasurfaces have provided unprecedented capacities in controlling the abrupt phase shifts, amplitude, and polarization of scattered EM waves based on surface engineered metallic patterns [5], and were recently promoted for ultrathin invisibility skin cloak by wrapping it over a target [6]. It provided an alternative paradigm toward rendering arbitrarily shaped scatterers invisible. The crucial principle is that the complex wavefront of intensity and polarization scattered by the metaplate can be reconstructed exactly the same as if it was deflected from arbitrarily predicted object, typically a flat ground. After this groundbreaking attempt, other forms of ultrathin cloaks have been addressed recently, such as amplitude-phase preserved cloak [7], illusion cloak [8], external cloak [9], and full-polarization cloak [10]. Of particular importance, 3-dimensional (3D) pyramid cloak has been realized by combining conformal and 3D printing technique [10], readily extendable to arbitrary complex structures and platforms. To summarize, we term the above cloaking paradigm as the first-generation technique for clarity, as illustrated in the evolutionary path in the top panel of Fig. 1. Although different mechanisms have been comprehensively developed for invisibility in terms of absorptions, diffusive scatterings, perfect transmissions, or illusions, the physical size to be concealed under the cloaks is extremely limited. Most importantly, the scattering features are passively fixed whose operation frequency and functionality cannot be dynamically switched once they are fabricated, making the fast and active stealth adaptive to the complex and dynamic EM environment a pressing task. This is especially true for the scenarios in complex electronic countermeasure where frequency agility, polarization agility and beam shifting are major concerns.

Fortunately, to address the aforementioned scientific bottlenecks inherent in the first generation, tunable meta-arrays have emerged with a reconfigurable and on-demand manner using active components [11,12], ferrite materials, and active materials including phase-change materials, 2-dimensional (2D) materials, shape-memory materials, and liquid metals [13]. Such a framework has evolved to programmable meta-arrays [14] and exhibited unprecedented capability of EM wave control when voltages imposed on diodes of each pixel were typically controlled via a field-programmable gate array (FPGA) [15]. On the other hand, artificial intelligence (AI) in terms of deep learning has emerged as a powerful tool for EM design [16]. When programmable meta-arrays meet AI, reconfigurable intelligent surfaces (RIS) have emerged as a powerful platform to perform invisibility task well for large-scale on-demand and quasi-adaptive cloak [17] with multiple individual output voltages for dynamic surroundings, facilitating the second-generation invisibility cloaks [18].

Despite these exciting progressive developments, all aforementioned cloaks suffered from a serious issue: they should adhere to the targets (named contact-based cloaks hereafter) rather than being apart from each other with arbitrary distance (termed as noncontact cloaks). Such paradigm, taking absorptive coating or metamaterials as an example [13], was constrained to several critical shortcomings including arduous task for maintenance, increased aerodynamic forces, and impaired mobility for a high-speed aircraft. Moreover, it does not meet the ever-growing requirements of integrated radiation and scattering (IRAS) control since the cloak and object are unable to be decoupled. All the above physical limitations in contact-based cloaks necessitated the move to the latter noncontact stage.

In contrast, noncontact stealth, making unprecedented flexibility, represents a substantial breakthrough and milestone in this field. The origin of noncontact stealth can be dated back to long-range scattering control [19] and external cloak [9] where the object was closely distributed outside the complementary shell, and were optically canceled by each other. Such a concept was numerically verified by a programmable metasurface [15] placed at a distance away from a metallic elliptical cylinder to be cloaked. Despite these recent strongly related sporadic studies, they were only capable of anti-detecting and left the detecting uninvolved, essentially distinguishing them far from the concept of noncontact cloaking established here. To clarify the disruptive concept, we define the cutting-edge innovative noncontact cloaks as third-generation stealth technologies. Essentially, such emerging strategy is also appropriate to be called supportive stealth where the external cloak functions as a carry-on system or equipment along with people. As of today, the latter noncontact supportive invisible metadevices are rarely reported, and their design is extremely complicated and thus remains elusive. Therefore, it is necessary to cast a holistic outlook for various state-of-the-art techniques to facilitate such a meta-cloak.

## Results

The conceptual third-generation noncontact stealth framework using the supportive RIS (SRIS) we envisioned is portrayed in the bottom panel of Fig. 1. As can be seen, the transformative paradigm is typically composed of several FPGA-driven active SRIS cloaks deployed far away from the targets and equipped with AI [18], in terms of various microcomputers, sensors, and actuators. To be more specific, the integration mechanism of several advanced technologies will be involved, such as IRAS or in other words integrated detection and anti-detection [20], integrated sensing and communication (ISAC), multi-SRIS deployments, instantaneous sensing of ambient parameters, and calculation of target scattering, agile generation of ambient-based scattering patterns with appropriate intensity and phase, and quick evaluation of invisibility. Explicit details about the physical principles of how these components work together to achieve noncontact invisibility are discussed in Methods. The key principle behind involves destructive interference of EM scattering between highly maneuverable cooperative targets and noncontact adaptive SRIS cloaks within specific angular domains. The precision of destructive interference to cancel out EM scattering from targets is associated with temporal alignment of target and SRIS echoes, and size and coding states of SRIS (see the “Construction of datasets and ANN models for AI control” and “System latency analysis and destructive interference precision” sections). Therein, it is essential for SRIS cloaks to quickly generate a predefined, real-time EM scattering within precise time synchronization by controlling its system processing latency. Therefore, the target's radar cross-section (RCS) is diminished or its scattering is blended with the surrounding background, and thus, the cooperative targets from our side are protected from being detected by a hostile radar system. Besides, such an SRIS system is also capable of adaptive control over frequency, polarization, waveform, and beam steering. Therein, multisensor systems are employed for multidimensional sensing of diverse parameters encompassing polarization, frequency, and direction of arrival (DOA) of enemy radar signals, EM characteristics of cooperative targets, as well as relative position and velocity among radar, targets, and SRIS cloaks (see the “Construction of datasets and ANN models for AI control” section). These acquired sensing data are then input into a pre-trained artificial neural network (ANN) model to rapidly estimate corresponding scatterings of several cooperative targets, which are subsequently assisted in generating counteracting scatterings of the SRIS (see the “Construction of datasets and ANN models for AI control” section). Finally, reflected amplitudes and phases are mapped out point by point over the meta-array aperture, and are eventually carried out as different voltages controlled via FPGA. To enhance compatibility across different radar bands, an ultra-wideband reconfigurable SRIS integrated with a frequency sensing module is adopted to provide broadband response typically covering multiband radar signals of S/C/X/Ku bands (2 to 18 GHz). Moreover, by leveraging sensors to perceive self-changes of targets (velocity, position, attitude, size, etc.) and radar (polarization, frequency, waveform, modulation scheme, DOA, etc.) and external environmental changes (temperature, air pressure, humidity, etc.), the SRIS also enables the instantaneous synthesis of the required scattering fields based on ANN and the configuration of the pixel-related amplitude and phase over the aperture to compensate for the corresponding distortions, and thus makes the system adaptive to those changes (see the “Construction of datasets and ANN models for AI control” and “System latency analysis and destructive interference precision” sections). Regarding multitarget cloaking scenarios, waveform orthogonalization and multiplexing EM wave control of SRIS such as spatial/frequency/polarization multiplexing enable simultaneous multibeam manipulations, and thus ensure channel interference suppression during concurrent detection, tracking, and cloaking of multiple targets. Most importantly, the blind spot of invisibility can be eliminated through joint deployment of several SRISs and by automatically altering orientations of them based on the negative feedback of intelligent microsystems (see the “Multi-SRIS deployments” section).

## Discussion

To sum up, a new generation of noncontact supportive cloak by using surface engineering is anticipated to empower future developments in interdisciplinary fields between optics, information, communication, computation, mechanics, and automatic control. The fast development of AI [18], sophisticated multiplexing EM wave control with vast capacity channels [21], multidimensional perception [22], and supercomputing capability of microsystems takes metasurface cloak to a plateau, over which cloaks can be engineered in vast real-time scenarios across highly maneuverable targets as well as time-sensitive targets, and in multidomain regimes including land, sea, and air. Moreover, multiphysics associated with EM effects, thermal dynamics, mechanical deformation, fluid, structural shape morphing, and force control should also be considered for future extreme environments without compromising mobility of the targets (see the “Analysis of multiphysics coupling pathways” section). Last but not the least, time coding strategy can also be introduced for deception cloak in velocity [23] and high-resolution range profile [24]. In addition to the new concept brought up in academia, the SRIS would be more promising in a myriad of industry application scenarios for the sake of its good technical adaptability. Of particular importance and epitome are the military stealth equipment and civilian EM compatibility protection. Therein, the high power intensity and short reaction time of SRIS should be 2 critical challenges for industrialization, especially for IRAS control and power management when deployed on drones (see the “IRAS control and power management of the SRIS platform” section). Moreover, its intellectualization and systematization level are also other key bottlenecks. Specifically, the ISAC, waveform integration, and time-division and frequency-division multiplexing should be further considered to avoid prohibitively low integration, spectrum mutual interference, and waste of resources [25]. Nevertheless, we believe that these challenges and bottlenecks are bound to be fully addressed one by one with the coordinated efforts of radar, communication, and antenna experts; optics scientists; AI researchers; and multiphysics and materials specialists.

## Methods

### Construction of datasets and ANN models for AI control

In the proposed noncontact stealth framework, the key to realize supportive stealth lies in rapidly acquiring the amplitude-phase patterns of SRIS based on sensing data, enabling the FPGA to drive the SRISs to rapidly generate the predefined, real-time EM scattering behavior by controlling different voltages. However, due to the complex interferences among the detection radar, cooperative targets, SRISs, and the environment, it is difficult to directly acquire the mapping from sensing data to SRIS amplitude-phase patterns within the limited time via specific numerical derivations or empirical physical models. To engineer this, we adopt here an ANN model in microcomputers to provide real-time input for the control action of FPGA.

Construction of an accurately labeled dataset is a crucial prerequisite for ANN. Meanwhile, for a specific set of sensing data, the amplitude-phase patterns that meet noncontact stealth requirements are difficult to obtain by experiments or EM simulations, especially in complex scenarios with multiple cooperative targets and multiple SRISs. Therein, determination of the amplitude-phase patterns of SRISs for cooperative target invisibility can be envisioned as an optimization issue, and the construction of the dataset can be accomplished by iterative computations.

**Fig. 2.**
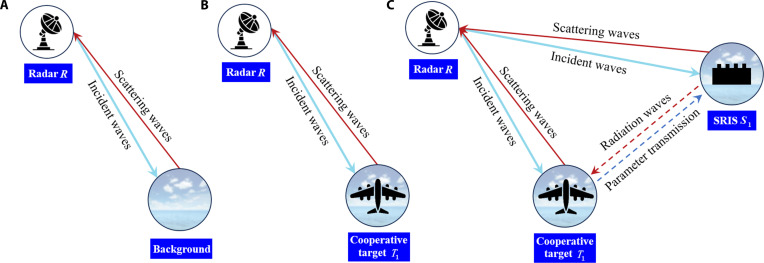
Scattering schematic at 3 scenarios of (A) background only, (B) a cooperative target in background, and (C) the noncontact supportive stealth containing single SRIS and one cooperative target in background. The total scattered field is indicated as *E*_0_, *E*_1_, and *E*_2_, respectively.

Specifically, we construct their mapping relationship in a stepwise manner from simplicity to complexity. Firstly, we consider the background-only scenario as illustrated in Fig. 2A, where a detected wave is emitted from radar *R* located at (*x*_0_, *y*_0_, *z*_0_). The total scattering field $E_{0}=F\left(G_{r},G_{b}\right)$ received by the radar corresponds to the background field; here, *F* represents the mapping function associated with sensing data *G*_r_ (radar parameters such as polarization, frequency, waveform, modulation scheme, and DOA) and *G*_b_ (background parameters set covering the temperature, atmospheric pressure, and humidity of detection area). In the second scenario as illustrated in Fig. 2B, cooperative target *T*_1_ appears at (*x*_1_, *y*_1_, *z*_1_), resulting in the total scattering field of $E_{1}=F\left(G_{r},G_{b},\left[\vec{RT_{1}},G_{T_{1}}\right]\right)$, where $G_{T_{1}}$ denotes target parameters of *T*_1_ such as velocity, position, attitude, and size, and $\vec{{\mathrm{RT}}_{1}}$ represents position vector from *R* to *T*_1_, associating with coordinate parameters as

$$\begin{array}{c} \left\{\begin{array}{c} \left|\vec{{\mathrm{RT}}_{1}}\right|=\sqrt{{\left(x_{1}-x_{0}\right)}^{2}+{\left(y_{1}-y_{0}\right)}^{2}+{\left(z_{1}-z_{0}\right)}^{2}}\\ \;\sin\theta_{1}=\frac{\sqrt{{\left(x_{1}-x_{0}\right)}^{2}+{\left(y_{1}-y_{0}\right)}^{2}}}{\sqrt{{\left(x_{1}-x_{0}\right)}^{2}+{\left(y_{1}-y_{0}\right)}^{2}+{\left(z_{1}-z_{0}\right)}^{2}}}\\ \sin\;\varphi_{1}=\frac{x_{1}-x_{0}}{\sqrt{{\left(x_{1}-x_{0}\right)}^{2}+{\left(y_{1}-y_{0}\right)}^{2}}} \end{array}\right. \end{array}$$ (1)

**Fig. 3.**
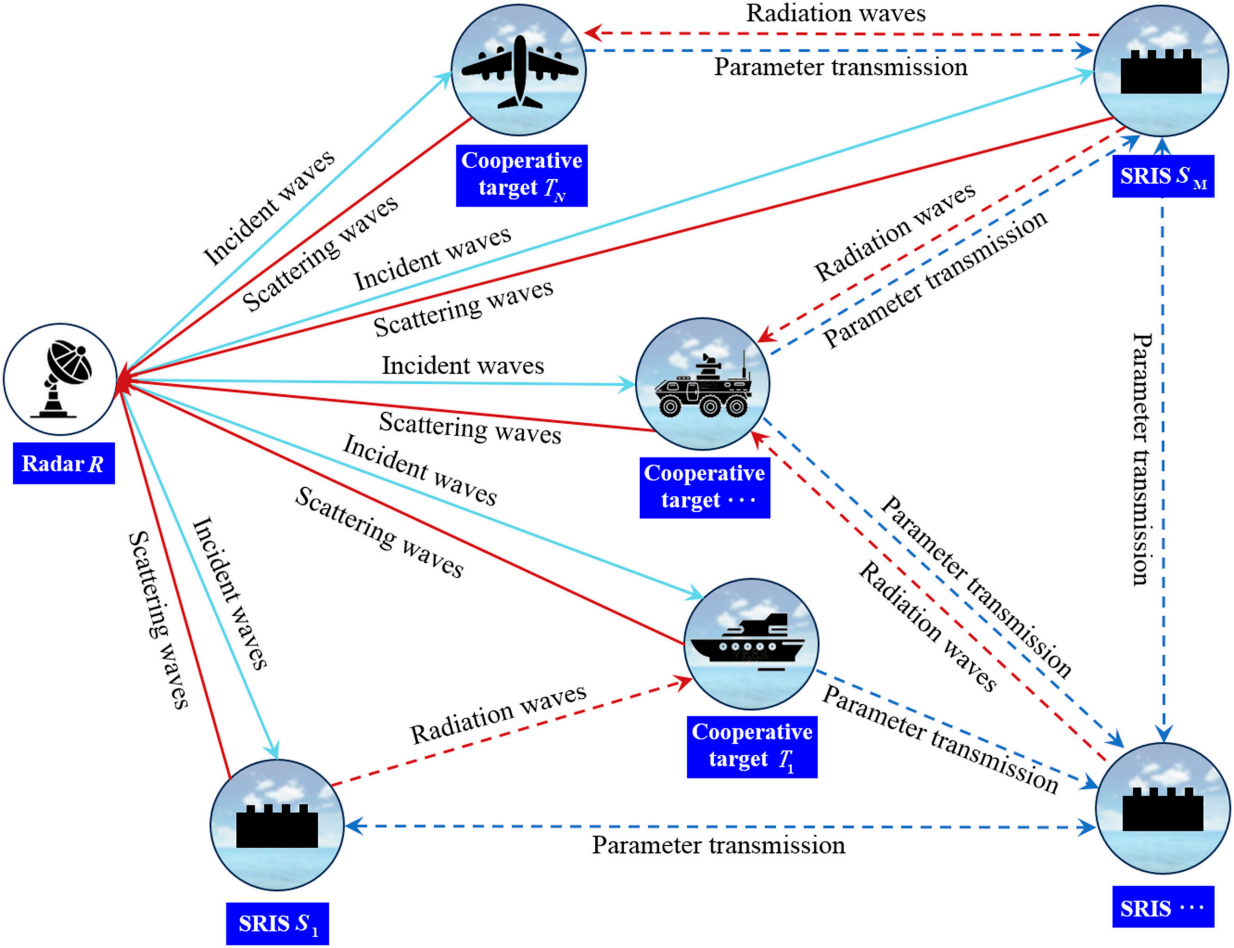
Scattering schematic of the noncontact supportive stealth for multiple cooperative targets of *N* by deploying multiple SRISs. The total scattered field is indicated as *E*_3_.

Here, *θ*_1_ and *φ*_1_ are the elevation and azimuth angle of incident wave received by *T*_1_, respectively. To achieve stealth for *T*_1_ in the third scenario of Fig. 2C, SRIS *S*_1_ at (*x*_2_, *y*_2_, *z*_2_) generates canceling scattering waves based on amplitude-phase pattern *P*_1_, such that the total scattering field $\begin{array}{c} E_{2}=F\left(G_{r},G_{b},\left[\vec{{\mathrm{RT}}_{1}},G_{T_{1}}\right],\left[\vec{{\mathrm{RS}}_{1}},P_{1}\right]\right) \end{array}$ resembles the background scattering field *E*_0_. Finally, we can achieve stealth for *N* cooperative targets by deploying *M* SRISs collaboratively to modulate the total scattered field $\begin{array}{c} \;E_{3}=F\left(G_{r},G_{b},{\left[\vec{{\mathrm{RT}}_{n}},G_{T_{n}}\right]}_{n=1}^{N},{\left[\vec{{\mathrm{RS}}_{m}},P_{m}\right]}_{m=1}^{M}\right) \end{array}$ , as shown in Fig. 3. Note that the SRIS is also capable of performing target detection, tracking, and communication by controlling beam scanning of SRIS.

Based on the above analysis, the optimization of amplitude-phase patterns of SRISs each composed of *u* meta-atoms (each with *c* coding states) can be expressed as

$$\begin{array}{c} \left\{\begin{array}{c} \min\;\left|E_{0}-F\left(G_{r},G_{B},{\left[\vec{RT_{n}},G_{T_{n}}\right]}_{n=1}^{N},{\left[\vec{{\mathrm{RS}}_{m}},P_{m}\right]}_{m=1}^{M}\right)\right|\\ s.t.\;P_{m}=\left[p_{m,1},\cdots ,\;p_{m,j},\cdots ,\;p_{m,u}\right],p_{m,j}\in \left\{1,\cdots ,\;c\right\} \end{array}\right., \end{array}$$ (2)

However, there remain 2 issues in optimization due to large-scale parameters induced by multiple cooperative targets and SRISs: (a) global optimal solution is difficult to obtain within limited time, and (b) *F* is difficult to build via specific numerical derivation or empirical physical models.

Thereafter, we adopt a local optimization strategy to obtain an approximate optimal solution fulfilling the stealth requirements, and use an ANN model to construct *F*. Specifically, commonly used algorithms such as topology optimization or heuristic algorithms are feasible in the former case. For ANNs, we introduce a graph neural network (GNN) to deal with the data of variable cooperative targets and SRISs manifesting different dimensions since GNN imposes no constraints on input dimensions. Specifically, design of the GNN_1_ involves the following 4 aspects. (a) Regarding the training dataset, inputs of GNN_1_ are sensing data (*G*_r_, *G*_b_, ${\left[G_{T_{n}}\right]}_{n=1}^{N}$), amplitude-phase patterns, and position vectors, while the output is the total scattered field. Since the computational logic of GNN_1_ is designed based on graph structures, the inputs of GNN_1_ need to be converted into graph-structured data, mainly consisting of nodes, edges, and their features [26]. Here, each SRIS or cooperative target is treated as a node, and sensing data or amplitude-phase patterns are treated as node features. Since the scattered fields of different entities are mutually coupled, there is an edge between any 2 nodes specified above, and their position vectors are defined as the edge feature. Moreover, the above sensing data, amplitude-phase patterns, position vectors, and corresponding total scattering field are collected via repeated experiments, and are split in a ratio of 8:2 for training and testing. (b) Regarding the network architecture, the message passing layer is a fundamental component of GNN_1_, mainly consisting of 2 feature extraction modules for any node. To ensure the performance of GNN_1_, the feature extraction module is designed with 5 fully connected layers, each followed by a rectified linear unit activation function. The former module aggregates the features of neighboring nodes and edges into a single vector, while the latter updates its representation based on this vector and its own features. (c) Regarding the model training, we introduce mean-square error (MSE) as the loss function, and incorporate physical constraints such as Maxwell’s equations and thermodynamic equations to alleviate the overfitting of GNN_1_ to the training dataset. Moreover, Adam optimizer is adopted for accelerating model convergence. (d) Regarding performance indicators, mean prediction error $e_{\mathrm{av}}=\frac{1}{k}\sum_{i=1}^{k}e_{i}$ is defined to quantify the accuracy, where *e_i_* is the error between the *i*th predicted value and the true value, and *k* is the number of samples in the test set.

**Fig. 4.**
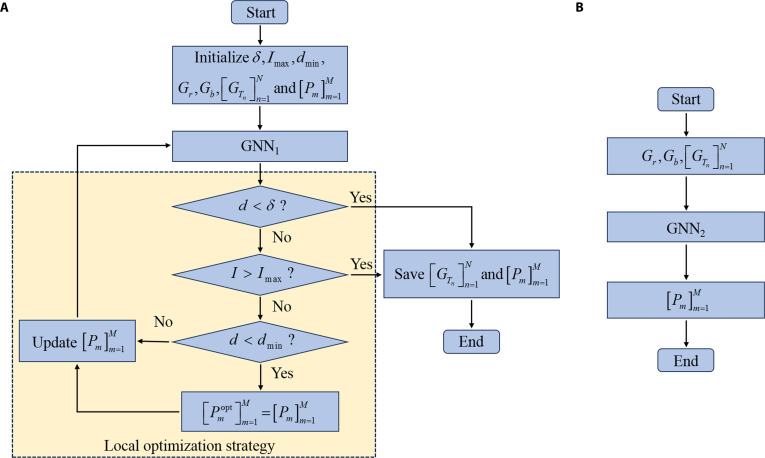
(A) Construction of single-round mapping record for sensing data and amplitude-phase patterns using GNN_1_ and local optimization strategy, and (B) prediction of amplitude-phase patterns using GNN_2_. Here, the local optimization strategy is used to update the amplitude-phase patterns while GNN_1_ is adopted to predict the total scattering field. Note that the dataset for training GNN_2_ is formed by repeating the single-round process in (A) to acquire a vast array of mapping records.

Afterwards, the procedures for acquiring sensing data and amplitude-phase patterns that meet noncontact stealth requirements are summarized in Fig. 4A. Firstly, randomly generate a group of *G*_r_, *G*_b_, ${\left[G_{T_{n}}\right]}_{n=1}^{N}$, and $\;{\left[P_{m}\right]}_{m=1}^{M}$, and define key parameters (threshold *δ*, maximum number of iterations *I*_max_, and minimum difference *d*_min_ between *E*_3_ and *E*_0_) for local optimization. Secondly, calculate the total scattered field *E*_3_ via GNN_1_. If difference *d* = *E*_3_ − *E*_0_ is less than *δ*, save the ${\left[G_{T_{n}}\right]}_{n=1}^{N}$ and $\;{\left[P_{m}\right]}_{m=1}^{M}$. Otherwise, determine whether the current iteration number *I* exceeds *I*_max_. If *I* > *I*_max_, save the optimal amplitude-phase patterns ${\left[P_{m}^{\mathrm{opt}}\right]}_{m=1}^{M}\;$. If *I* < *I*_max_, determine whether *d* is less than the current *d*_min_. If *d* < *d*_min_, set ${\left[P_{m}^{\mathrm{opt}}\right]}_{m=1}^{M}={\left[P_{m}\right]}_{m=1}^{M}$; otherwise, the original ${\left[P_{m}^{\mathrm{opt}}\right]}_{m=1}^{M}\;$ remains unchanged. Finally, update $\;{\left[P_{m}\right]}_{m=1}^{M}$ based on ${\left[P_{m}^{\mathrm{opt}}\right]}_{m=1}^{M}\;$ and re-input it into the GNN_1_. Notably, the mapping dataset between sensing data and amplitude-phase patterns will be efficiently constructed by repeating the aforementioned method since optimized patterns can be rapidly validated by GNN_1_. In the following, we construct a new GNN_2_ for predicting amplitude-phase patterns as shown in Fig. 4B. Different from the previous GNN_1_, GNN_2_ adopts graph-structured sensing data (node feature is 2 neighboring cooperative targets) as its input and graph-structured pattern data (node feature is 2 neighboring SRISs) as its output. Based on the feature extraction module of GNN_1_, a batch normalization layer is used before each fully connected layer to prevent the overfitting of GNN_2_ to the training dataset. Note that the inference time of GNN_2_ should be less than 50 ms to ensure the fast response of SRISs. Otherwise, model compression technologies will be introduced to reduce the model parameters of GNN_2_.

Meanwhile, we introduce adaptive mechanisms to deal with unknown scenarios in complex electronic warfare environments. Before GNN_2_ inference, the similarity between actually received and existing sensing data is calculated. If similarity exceeds pre-defined data deviation threshold, SRIS no longer adopts the predicted amplitude-phase patterns from GNN_2_, but instead obtains them based on GNN_1_ and the local optimization strategy in Fig. 4A. Moreover, received data are continuously merged with existing data to maintain the effectiveness of GNN_2_, enabling real-time parameter updates for the pre-trained model based on online learning. Specifically, the feature extraction module of GNN_2_ is divided into 2 parts, and we freeze the parameters of one part while only updating the other when retraining GNN_2_. This method alleviates the forgetting of past knowledge by the model while enabling it to quickly learn the features of new unknown signals.

### System latency analysis and destructive interference precision

#### Time synchronization and latency analysis

**Fig. 5.**
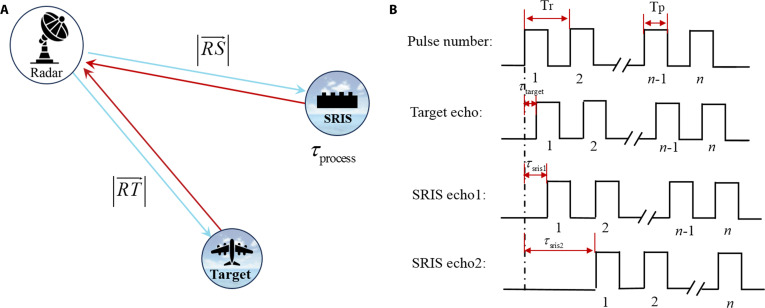
Schematic illustration of time synchronization and latency analysis. (A) Geometric relationship and (B) echo pulses of radar, target, and SRIS.

To analyze the feasibility of canceling a target echo using the echo from SRIS, a geometric model associated with relative positions of radar, target, and SRIS must be established. As illustrated in Fig. 5A, the model defines 2 core position vectors: $\vec{\mathrm{RT}}$ representing the vector between radar and target, and $\vec{\mathrm{RS}}$ indicating that between radar and SRIS. These distances ($\left|\vec{\mathrm{RT}}\right|$ and $\left|\vec{\mathrm{RS}}\right|$) directly determine the time delay of different echoes for radar signals, i.e., the target echo experiences an EM propagation delay of $\tau_{\text{target}}=2\left|\vec{\mathrm{RT}}\right|/c$, while the SRIS echo undergoes a different path plus internal processing. This results in a total delay of $\tau_{\text{SRIS}}=2\left|\vec{\mathrm{RS}}\right|/c+\tau_{\text{process}}$, where $\tau_{\text{process}}$ denotes the system processing latency of SRIS and *c* is the speed of light.

The relative arrival times of these periodic echoes, which are critical for achieving coherent cancellation, are consequently illustrated in the echo pulse diagram shown in Fig. 5B. From these temporal relationships, the conditions for achieving overlap of radar pulse width $T_{p}$ and, thus, coherent cancellation between the target and SRIS echoes can be determined. This leads to 2 principal scenarios: (a) $|\tau_{\text{SRIS}}-\tau_{\text{target}}|\leq T_{p}$ with overlapping of target and SRIS echoes. In such a case, target echoes can be fully ($\tau_{\text{SRIS}}=\tau_{\text{target}}$) or partially ($\tau_{\text{SRIS}}\approx \tau_{\text{target}}$) cancelled by the SRIS echoes. To fulfill this condition, $\left|\vec{\mathrm{RS}}\right|$ and $\tau_{\text{process}}$ should be carefully designed. (b) For a synthetic aperture radar or a radar employing the pulse integration technique without perfect synchronization due to practical constraints to $\left|\vec{\mathrm{RT}}\right|-\left|\vec{\mathrm{RS}}\right|$ and $\tau_{\text{process}}$, its target detection performance will be well reduced given $|\tau_{\text{SRIS}}-\tau_{\text{target}}-\left(m-1\right)T_{r}|\leq T_{p}$; even the former (*m−*1) target echoes cannot be cancelled by SRIS. In this scenario, target echoes beyond the *m*th one (m≥2) can also be cancelled by SRIS echoes via carefully designing $\vec{\mathrm{RS}}$ and $\tau_{\text{process}}$. Here, $T_{r}$ denotes the pulse repetition interval, and *m* is the number of pulse index. Indeed, the 2 scenarios described above can be unified into a single general condition: $|\tau_{\text{SRIS}}-\tau_{\text{target}}-\left(m-1\right)T_{r}|\leq T_{p}$, with m≥1. In a word, the temporal alignment between SRIS and target echoes collectively determine the effectiveness of cancellation.

Notably, achieving aforementioned synchronization condition depends critically on $\tau_{\text{process}}$. This key controllable parameter can be divided into 3 primary parts in practice: (a) signal acquisition latency with a nanosecond-scale delay originating from SRIS and receiving chain, (b) intelligent processing latency comprising both microsecond-scale signal preprocessing and millisecond-scale AI inference delays, and (c) SRIS reconfiguration latency governed by microsecond-scale switching of SRIS’s electronically controlled elements. In summary, system processing latency $\tau_{\text{process}}$ is a critical factor affecting precise time synchronization, which necessitates a system-level optimization approach for the above 3 latency parameters.

#### Precision analysis of destructive interference

**Fig. 6.**
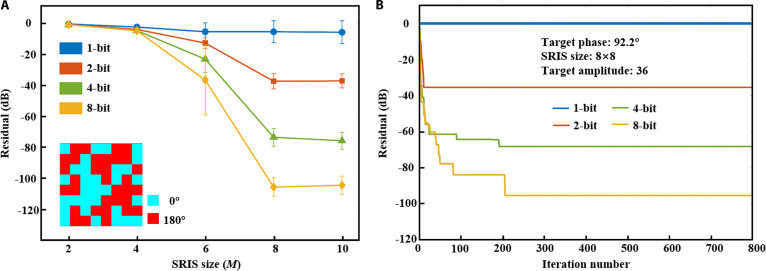
Cancellation performance and convergence tendency using the particle swarm optimization (PSO) algorithm. The calculation is performed for a target with a fixed scattering amplitude of *A*_t_ = 36 and random phase *φ*_t_, and an SRIS with unit-amplitude meta-atom and 2*^b^* coding states. Results are derived from 800-time Monte Carlo simulations using PSO to determine the optimal phase distributions of SRIS. (A) Residual *r* as a function of *M* and *b* of SRIS. The subgraph shows optimized phase distributions for the case of *M* = 8 and *b* = 1 with a target phase *φ*_t_ =92.2°. (B) Convergence curves of PSO with *M* = 8 and *b* = 1, 2, 4, and 8.

Following the above analysis of time synchronization conditions between SRIS and target echoes, this section will further explore the precision of destructive interference, which determines the cancellation effectiveness. Assuming the SRIS and target echoes are synchronized in time, the precision of the destructive interference produced by SRIS depends on its amplitude and phase modulation accuracy. Define the complex *E*-field amplitudes of target and SRIS as $E_{\text{target}}=A_{t}e^{j\varphi_{t}}$ and $E_{\text{SRIS}}=A_{s}\;e^{j\varphi_{s}}$, respectively. To achieve perfect cancellation, the total complex amplitudes $E_{\text{total}}=E_{\text{target}}+E_{\text{SRIS}}$ should be zero, which requires equal amplitudes ($A_{s}=A_{t}$) and opposite phases ($\varphi_{s}=\varphi_{t}+\left(2n+1\right)\pi ,n\in \mathbb{Z}$). Therein, the amplitude and phase modulation precision of SRIS are determined by 2 key parameters: the number of meta-atoms *M* and phase coding states $2^{b}$ (*b* bits). The effectiveness of cancellation can be characterized by residual in dB defined as $\left|r=20\log10\left(E_{\text{total}}/E_{\text{target}}\right)\right|$. As illustrated in Fig. 6, it demonstrates that the cancellation performance of SRIS is significantly improved as *M* and *b* increase. Specifically, the residual remains large (e.g., ≥−10 dB) when *M* ≤ 4, and *M* = 6 with *b* ≤ 2. In contrast, an extremely smaller residual (e.g., ≤−100 dB) is expected when *M* ≥ 8 with *b* = 8, indicating the realization of high-precision destructive interference.

### Multi-SRIS deployments

**Fig. 7.**
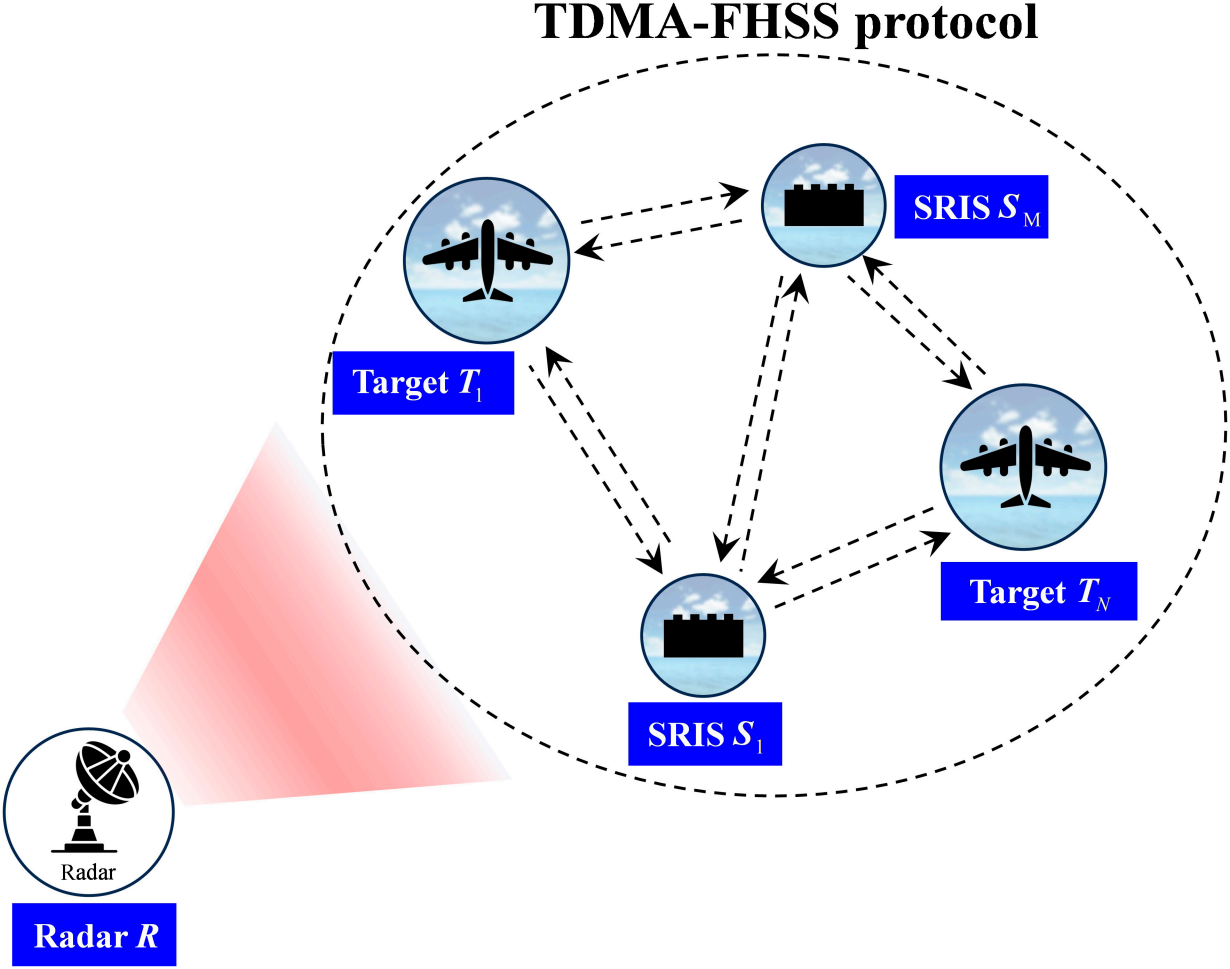
Illustration of the dedicated multi-SRIS coordination network (dashed oval), featuring distributed peer-to-peer links, synchronization signaling, and data exchange via a TDMA-FHSS protocol among several SRISs (*S*_1_, *S*_2_, …, *S*_M_) and cooperative targets (*T*_1_, *T*_2_, …, *T*_N_).

To ensure precise coordination and survivability among multiple SRISs, a distributed peer-to-peer cooperative mechanisms is adopted, as shown in Fig. 7. Each SRIS is equipped with a local microcomputer and connects directly to others via a high-reliability, low-latency wireless ad-hoc network. Therein, sensing data, status information, and cooperative commands are exchanged through the network. This network operates on a dedicated time division multiple access (TDMA)-based data transmission protocol with frequency-hopping spread spectrum (FHSS) [27], guaranteeing precise time synchronization and robust anti-jamming performance. Meanwhile, initial attitude synchronization among several SRISs (*S*_1_, *S*_2_, …, *S*_M_) and cooperative targets (*T*_1_, *T*_2_, …, *T*_N_) is required to guarantee rough alignment. Further accuracy improvement of beam direction with errors less than 1° is fulfilled by controlling electric beam scanning of SRIS for target detection, tracking, and communication.

**Fig. 8.**
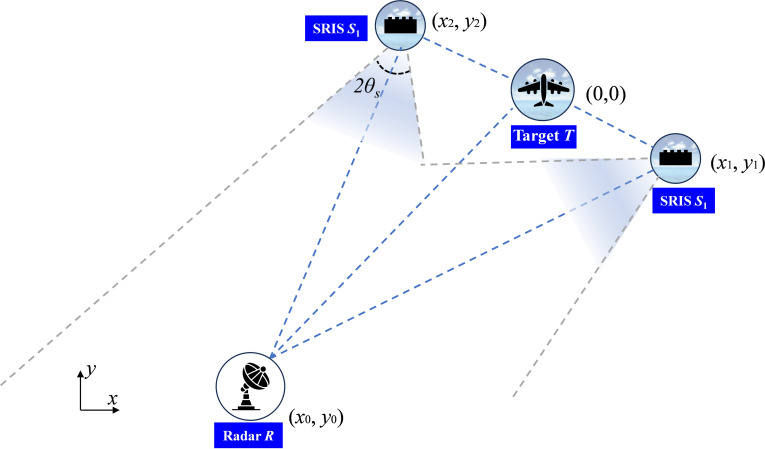
Schematic diagram of dual-SRIS deployment for aerial targets on 2D plane. Two SRISs are deployed on 2 sides of the cooperative target, respectively. Each SRIS aims for the radar with an effective scattering coverage area of 2*θ_s_*.

To reduce the blind spots of invisibility, optimization of multi-SRIS deployment should be further conducted. Firstly, the blind spot of invisibility is defined as the spatial regions where an enemy radar retains the capability to effectively detect the cooperative target after the SRISs have been deployed. In other words, this region corresponds to the area beyond the SRIS effective coverage region (ECR). Then, we define the criterion for blind-spot elimination as suppression of the target’s RCS within 2 dB compared to the RCS of the background. The optimal number of SRISs depends on practical deployments, threat in the environment, and target parameters. For a preliminary quantitative analysis, we investigate a simplified scenario involving aerial targets on a 2D plane (see Fig. 8): a radar at (*x*_0_, *y*_0_) and a cooperative target at (0, 0) defended by 2 cooperative SRISs at (*x*_1_, *y*_1_) and (*x*_2_, *y*_2_), respectively.

Assuming that the radar moves only along the *x* axis, the ECR of each SRIS on the line *y* = *y*_0_ can be derived geometrically. The joint ECR of the dual-SRIS system thus corresponds to the union of 2 individual ECRs. On this basis, the ECR of *I_i_* for the *i*th SRIS (*i* = 1, 2) can be written as Eqs. 3A and 3B, respectively, when $y_{0}>y_{i}$ and $y_{0}<y_{i}$.

$$\begin{array}{c} I_{i}=\left\{\begin{array}{c} \left[x_{i,+},\;x_{i,-}\right],\alpha_{i}-\theta_{s}>0\&\alpha_{i}+\theta_{s}<\pi \\ \left[x_{i,+},\;+\infty \right),\alpha_{i}-\theta_{s}\leq 0\\ \left(-\infty ,\;x_{i,-}\right],\alpha_{i}+\theta_{s}\geq \pi \end{array}\right. \end{array}$$ (3A)

$$\begin{array}{c} I_{i}=\left\{\begin{array}{c} \left[x_{i,-},\;x_{i,+}\right],\alpha_{i}+\theta_{s}<0\&\alpha_{i}-\theta_{s}>-\pi \\ \left[x_{i,-},\;+\infty \right),\alpha_{i}+\theta_{s}\geq 0\\ \left(-\infty ,\;x_{i,+}\right],\alpha_{i}-\theta_{s}\leq -\pi \end{array}\right. \end{array}$$ (3B)

Here, $x_{i,\pm }=x_{i}+\left(y_{0}-y_{i}\right)\cot\left(\alpha_{i}\pm \theta_{s}\right)$ and $\alpha_{i}=\text{atan}2$ $\left(y_{0}-y_{i},x_{0}-x_{i}\right)$.

To quantitatively determine the optimal SRIS deployment by minimizing the blind-spot regime (maximizing ECR), we take following scenario as an example. The radar, cooperative target, and 2 SRISs are located at *R*(−20 km, −50 km), *T*(0,0), *S*_1_(−10 km, 0), and *S*_2_(10 km, 0), respectively. Each SRIS is deployed within a 20-km communication range from the cooperative target with an effective scattering coverage area of 2*θ_s_* = 60°. Under this configuration, the optimization model for ECR can be further defined as

maxI1∪I2s.t.x12+y12≤400x22+y22≤400 (4)

**Fig. 9.**
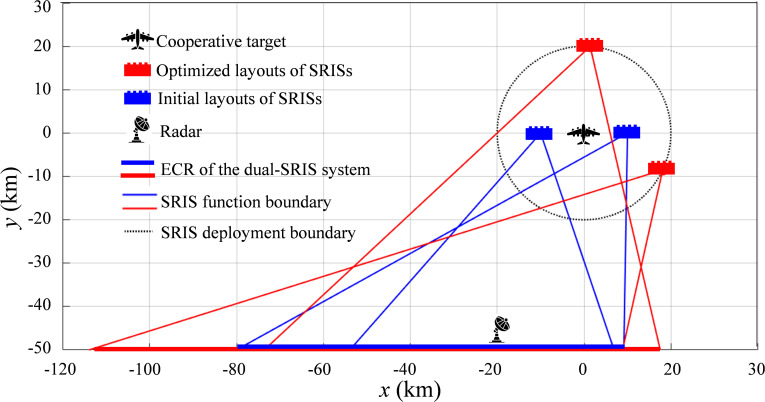
Blind-spot elimination illustration by comparing SRIS coverage area before and after an optimization of dual-SRIS deployment. Two SRISs are initially located at (−10 km, 0) and (10 km, 0) with numerically calculated ECR of [−80.07 km, 9.16 km] using Eq. 3B. Their positions are optimized as (1.40 km, 19.95 km) and (18.11 km, −8.48 km) through the SQP method with resulting ECR expanding to (−113.98 km, 17.54 km). Consequently, our optimized dual-SRIS deployment has reduced the blind spot by 42.29 km compared to its initial layout.

The initial blind-spot region can be calculated as (−∞, −80.07 km∪ $\left[9.16\;\mathrm{km},+\infty \right)$ according to Eqs. 3A and 3B. The deployment is then optimized using a multistart sequential quadratic programming (SQP) approach, with the objective of a minimized blind-spot region as $\left(-\infty ,-113.98\;\mathrm{km}\right]$ ∪ $\left[17.54\;\mathrm{km},+\infty \right)$ subject to communication-range constraint. Compared to the initial dual-SRIS location, the optimized dual-SRIS deployment reduces the blind-spot region by 42.29 km; see the blind-spot elimination by comparing the SRIS coverage area before and after the optimization shown in Fig. 9. This case study provides a preliminary numerical verification that multi-SRIS cooperative deployment can effectively reduce blind spots of invisibility. Moreover, such methodology is also applicable to ground-moving targets though it currently focuses on aerial targets.

**Fig. 10.**
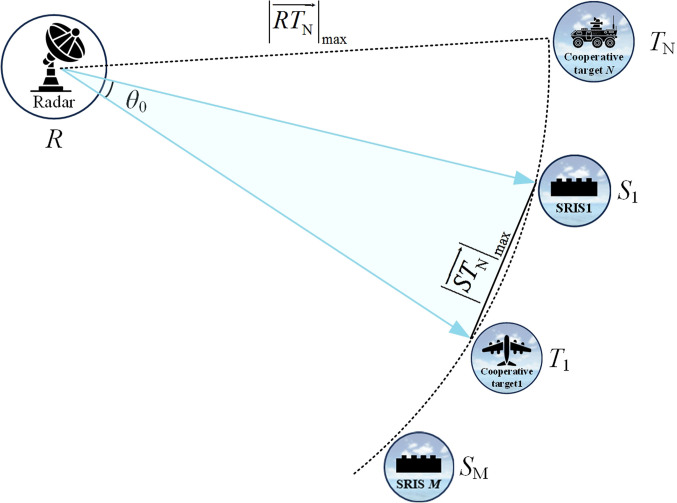
Effective operating distance between SRIS and target under multi-SRIS deployments.

Furthermore, the maximum effective operating distance is another essential index to ensure the stability of multiobjective operations in multi-SRIS deployments. To demonstrate the maximum operating distance between SRIS and the target, we consider a representative case for discussion; see the corresponding system model illustrated in Fig. 10. To achieve effective stealth protection, it is essential to ensure that both target and SRIS are located within the same main scanning beamwidth of hostile radar. Moreover, a fundamental geometric constraint of approximately equal distances of SRIS and target to the radar should also be fulfilled. The above constraints lead to following condition.

$$\left|\vec{{\mathrm{ST}}_{N}}\right|\leq 2{\left|\vec{{\mathrm{RT}}_{N}}\right|}_{\max}\cdot \sin\left(\theta_{0}/2\right)$$ (5)

Here, $\theta_{0}$ is the beamwidth of the hostile radar, ${\left|\vec{{\mathrm{RT}}_{N}}\right|}_{\max}$ is the maximum radar detection range, and $\left|\vec{ST_{N}}\right|$ represents the distance between SRIS and target. According to Eq. 5, the maximum effective operating distance ${\left|\vec{ST_{N}}\right|}_{\max}$ is influenced not only by our deployment but also by the system capability of hostile radar. The maximum detection range of hostile radar can be determined using the radar equation.

$$P_{r}=\frac{P_{t}G_{t}G_{r}\lambda^{2}\sigma }{{\left(4\pi \right)}^{3}R^{4}}$$ (6)

Here, we take an X-band search radar operating at 10 GHz as an example with a wavelength of 0.03 m, a transmit power *P*_t_ = 1,000 kW, an antenna gain of *G*_t_ = 35 dB, and a minimum detectable signal power of *S*_i min_ = −110 dBm. According to Eq. 6, ${\left|\vec{{\mathrm{RT}}_{N}}\right|}_{\max}$ can be formulated as

$${\left|\vec{{\mathrm{RT}}_{N}}\right|}_{\max}={\left[\frac{P_{t}G^{2}\lambda^{2}\sigma }{{\left(4\pi \right)}^{3}S_{i\min}}\right]}^{\frac{1}{4}}$$ (7)

Considering a target with standard RCS of *σ* = 1 m^2^, the maximum effective detection range is calculated as ${\left|\vec{{\mathrm{RT}}_{N}}\right|}_{\max}$ ≈ 150 km. Assuming the beamwidth of hostile radar *θ*_0_ ranging from 5° to 10°, ${\left|\vec{ST_{N}}\right|}_{\max}$ is roughly estimated to be 12 to 30 km based on the aforementioned geometric relationship described in Eq. 5.

### Analysis of multiphysics coupling pathways

**Fig. 11.**
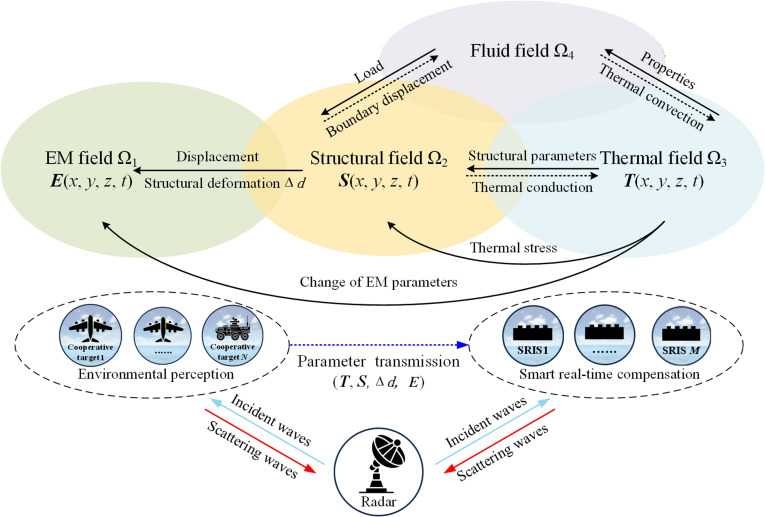
Schematic diagram of multiphysics coupling pathways to afford in-depth analysis of how extreme environments affect SRIS device performance.

As highlighted in the main text regarding future challenges, a comprehensive analysis of coupled EM, thermal, fluid, and structural fields is essential for strengthening the robustness of SRIS systems in terms of improving amplitude-phase control accuracy and sensor sensitivity in extreme complex environments. To systematically analyze interactions among different coupling forms, a schematic diagram of multiphysics coupling pathways and mitigation strategies is depicted in Fig. 11.

First, the analysis begins with thermal field, as temperature serves as a foundational driver of multiphysics coupling in the system. The influence of high temperature on SRIS performance mainly originates from 2 multiphysics coupling paths: (a) temperature-dependent material’s constitutive parameters that directly alter EM field distributions, and (b) thermal stress which modifies internal stress of structural field and may disturb the surrounding fluid field. Therefore, temperature variations can lead to changes in structural parameters and properties. These coupled effects at the material, EM, mechanical, and structural levels collectively degrade phase-control accuracy and sensor sensitivity by modifying both EM characteristics and spatial positions of array elements. Next, structural field couples with and influences the EM, thermal, and fluid fields. Under strong vibration, it primarily involves mechanical displacement of array elements and connection reliability damage. Such displacement thereby disrupts the geometric and phase coherence of the array, and ultimately degrades EM performance. From a multiphysics perspective, it also affects thermal conduction and thus induces thermal stress, which in turn alters the internal state of the structure field. These 2 aspects of change further propagate by influencing EM field distribution through structural deformation Δ*d* and modifying fluid velocity distribution through boundary displacement. Similarly, flow field influences thermal field distribution and structural internal stress through thermal convection and fluid load, respectively. Correspondingly, temperature variations and boundary displacements also alter the properties and boundary conditions of the fluid field. Finally, at the terminal of multiphysics coupling pathways, the impact of complex EM interference on the EM field of the SRIS device is fatal and fundamentally contributes to interactions among the 3 residual fields. Specifically, interference-induced vibrations lead to structural deformation and change of EM parameters, while accumulated EM energy further couples into and disturbs thermal and flow fields.

To engineer the above 3 issues in extreme environments (e.g., high temperature, strong vibration, and complex EM interference), synergistic solutions combining optimum design and adaptive control are required to comprehensively enhance the system’s stability under extreme environments. For the former optimum design, 3 strategies are proposed: (a) high-temperature-resistant materials to minimize deformation caused by thermal changes, (b) damping structures to reduce the impact of mechanical vibration, and (c) broadband meta-atoms with low sensitivity to incident angles to mitigate the effect of deformation, and coupled with an anti-interference network to enhance EM robustness. For latter adaptive control, real-time data on thermodynamic parameters, structural deformation, and EM parameters are acquired via sensors in SRIS. These data are processed by ANN for environmental perception, with the resulting output subsequently integrated into the control system to facilitate dynamical amplitude-phase compensation. This synergistic approach establishes mitigation strategies that extend from source suppression to real-time closed-loop compensation, thereby ensuring system stability.

### IRAS control and power management of the SRIS platform

**Fig. 12.**
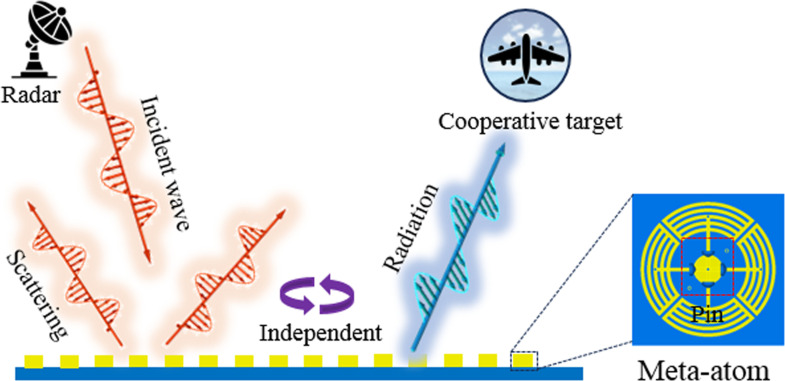
Schematic diagram of the SRIS platform with independent dual-mode radiation and scattering control. It switches between radiation mode (for cooperative target detection/tracking) and scattering mode (for stealth) by controlling diodes imparted in meta-atoms.

Unlike conventional cloaks, the SRIS platform composed of FPGA-driven PIN-diode-integrated metasurface and parallel-fed network supports the simultaneous control of both radiation and scattering modes. As shown in Fig. 12, it enables independent, dynamic, and decoupled phase modulation for each mode. In radiation mode, the platform actively generates highly directive beams capable of dynamic scanning via a fast beam-forming network and algorithm. It enables target detection and continuous tracking of high-mobility targets by quickly pointing SRIS beams to current and anticipated positions of targets. In scattering mode, the amplitude-phase-modulated SRIS generates a predefined, real-time EM scattering field to cancel that of cooperative targets within specific angular domains. This effectively reduces the RCS of the targets or enables them to blend with the surrounding environmental background. Through this mechanism, the total scattering received by hostile radar systems can approximate the environmental background scattering, thereby achieving protection for our cooperative targets. Moreover, the SRIS platform requires capabilities in multidimensional sensing and intelligent prediction to achieve self-adaptive radiation-scattering control. To engineer this, radar parameters, background parameters, and target parameters are captured through a multisensor system of targets (see Fig. 2 for details). These acquired sensing data are communicated to SRIS via beam alignment between them for the rapid estimation of scattering of multiple cooperative targets. In summary, the SRIS platform achieves dynamic target detection, tracking, and adaptive invisibility for cooperative targets through compound IRAS control, multidimensional sensing, and intelligent prediction.

The power consumption of the SRIS when deployed on drones can be addressed through the following 3 strategies: (a) low-power SRIS design by using complementary metal-oxide-semiconductor diodes and optimizing meta-atoms to reduce RF and bias currents, respectively; (b) novel energy utilization employing solar-powered drones with solid-state batteries, hydrogen fuel cells, flexible photovoltaics, and even wireless charging for continuous supply [28]; and (c) dynamic power management implemented via a multiplatform alternate mechanism, enabling full-power operation and on-demand precise-power mode. For example, it is indicated that a decline of bias current from 8.83 to 0.03 mA by meta-atom optimization can reduce total RIS consumption from 731.1 to 1.9 mW with negligible deterioration of power supply [29]. Note that the SRIS’s scattering can also be cancelled by targets according to the principle of reciprocity, making the SRIS platform self-protected and capable of survivability. Therefore, the low-consumption strategy of metasurfaces and new-energy active power together with dynamic power management considerably alleviate the constraints to energy source and the RCS management of the SRIS platform.

## Data Availability

All data needed to evaluate the conclusions of the study are present in the paper.
